# Physiological and Pathological Function of Serine/Arginine-Rich Splicing Factor 4 and Related Diseases

**DOI:** 10.1155/2018/3819719

**Published:** 2018-02-12

**Authors:** Wanyan Tan, Wei Wang, Qingfeng Ma

**Affiliations:** ^1^Department of Gastroenterology, Liyuan Hospital, Tongji Medical College, Huazhong University of Science and Technology, Wuhan 430077, China; ^2^Department of Clinical Laboratory, Puai Hospital, Tongji Medical College, Huazhong University of Science and Technology, Wuhan 430030, China; ^3^Medical Research Institute, Kanazawa Medical University, Ishikawa 920-0293, Japan

## Abstract

Serine/arginine-rich splicing factors (SRSFs) have one or two RNA recognition motifs in the N terminal and a serine/arginine-enriched domain in the C terminal. SRSFs are essential components of spliceosomes and are involved in alternative splicing, spliceosome assembly, mRNA export, and nonsense-mediated mRNA decay. The maintenance of cellular and tissue homeostasis relies on accurate alternative splicing, and various patterns of abnormal alternative splicing can cause different diseases. SRSF4 is associated with many physiological and pathological processes and has applications in the diagnosis and prognosis of specific diseases. In this review, we discuss knowledge of SRSF4 in physiological and pathological processes and highlight the applications of SRSF4 in the regulation of gene expression and associated diseases.

## 1. Introduction

In eukaryotes, RNA processing is necessary to produce mature mRNAs. This process includes three steps: (1) addition of a 5′ 7-methyl guanosine (m7G) cap (capping) to protect the growing RNA chain from degradation by nucleases; (2) addition of a 3′ poly-A tail (polyadenylation) to enhance the stability of mRNA and regulate mRNA transport to the cytoplasm; and (3) RNA splicing to remove noncoding sequences. RNA splicing is an essential posttranscriptional modification for precise translation of eukaryotic genes [1] and is catalyzed by spliceosome assembly from five small nuclear ribonucleic proteins (snRNPs) and numerous protein cofactors [2].

Serine/arginine-rich splicing factors (SRSFs) are the most important cofactors of snRNPs. There are 12 types of SR proteins in mammalian cells [3], all of which are structurally similar and contain one or two RNA recognition motifs (RRMs) and a serine/arginine (RS) rich domain [4]. SR proteins enter the nucleus through the functions of specific transport proteins [5] and predominantly localize in the nucleus [6, 7]. Moreover, SR proteins play important roles in posttranscriptional modifications [8]. During constitutive splicing, phosphorylated SR proteins mediate binding of the U1 snRNP to the 5′ splicing site, and polymerase II mediates binding of the U2 snRNP to the 3′ splicing site; U1 and U2 then interact to form a complex, recruit the U4/U6-U5 tri-snRNP to form spliceosomes, and undergo a series of rearrangements and splicing rounds to remove introns [9, 10].

Through alternative splicing, a single gene can encode multiple variant proteins with diverse biological functions, greatly enhancing the transcriptome complexity and diversity of proteins [11]. Regulation of alternative splicing is essential for the maintenance of cellular and tissue homeostasis, and various RNA binding proteins are involved in this process. Additionally, abnormal splicing can cause disorders associated with disruption of gene expression [12] and can promote the development of certain types of cancers [13, 14]. Heterogeneous nuclear ribonucleoproteins (hnRNPs) and SRSFs are the most important regulators of RNA splicing. For example, the *μ*2 protein of reovirus T1L represses the antiviral response of interferon-*α*/*β* and alters its localization to nuclear speckles through forming complex with SRSF2, thereby promoting reovirus T1L replication [15]. Additionally, infection with human papillomavirus 16 (HPV16) can cause anogenital cancer, and SRSF2 is essential for maintaining the stability of E6E7 mRNAs of HPV16, thereby promoting anogenital tumorigenesis [16]. The start codon of the human immunodeficiency virus- (HIV-) 1 accessory protein Vif is located at the downstream of HIV-1 noncoding exons 2/2b, and SRSF1, SRSF4, and SRSF10 bind to the HIV-1 exonic splicing enhancer (ESE2 and ESE2b). Then, after binding of heterogeneous U1 to the Vif start codon,* Vif* mRNA expression and HIV viral replication are increased within the host cells [17]. Moreover, SRSF1 has been implicated in neoplastic lung growth, cancer hyperplasia and metastasis, hypertension, and atherosclerosis [18]. SRSF1 and SRSF9 recruit *β*-catenin mRNA and enhance its expression, resulting in promotion of tumorigenesis by enhanced Wnt/*β*-catenin signaling [19]. SRSF5–7 are upregulated in small cell lung cancer (SCLC), and SRSF5 has diagnostic potential in SCLC and extrapulmonary pleural metastatic cancer [20]. Notably, SRSF2 is frequently mutated in chronic myelomonocytic leukemia and secondary acute myeloid leukemia derived from myelodysplastic syndromes or myeloproliferative neoplasms [21].

In this review, we discuss the physiological and pathological functions of SRSF4 and its relationship with diseases.

## 2. Physiological Function of SRSF4

### 2.1. Structure, Function, and Subcellular Localization of SRSF4

SRSF4 contains two RRMs and one RS domain. The RRM can directly contact RNA and determine the binding specificity of the RS domain. Additionally, phosphorylation of the RS domain can alter SRSF activity and localization, and the RS domain can also modulate protein interactions [3]. SRSF4 is mainly localized in the nucleus, where it participates in pre-mRNA splicing. Like some SRSFs, SRSF4 can also shuttle between the nucleus and cytoplasm and mediates mRNA export, stability, and translation through its shuttling activity [22].

### 2.2. SRSF4 Participates in the Splicing of Detained Introns (DIs) to Modulate Gene Expression

Splicing and transcription are coupled. For many years, noncoding introns were thought to be removed prior to transcriptional termination and polyadenylation of pre-mRNA [23]; however, abundant introns were detected within polyadenylated transcripts by in situ hybridization and high-throughput sequencing [24, 25], and these introns were then designated DIs. Subsequently, thousands of DIs have been identified. DIs can remain in the nucleus with half-lives of over 1 h and are insensitive to nonsense-mediated RNA decay [26]. Under normal conditions, transcripts with DIs are retained in the nucleus. Once cells are exposed to physiological stress (e.g., heat, osmotic shock, toxins, and compounds), splicing changes to protect cells against and adapt to the environmental stress; notably, the translation of noncoding DIs can be activated or inhibited under cell stress [26].

The activities of CDC-like kinases (CLKs) affect many DIs through modulation of SR protein phosphorylation. Some small compounds can inhibit CLK activity, and the splicing of DIs is altered following inhibition of CLK activity. SRSF4 is a CLK target, and its phosphorylation is dramatically shifted due to inhibition of CLK. This phosphorylation affects CB19-activated splicing and leads to DNA damage, which triggers changes in splicing, including that of DIs. CLK inhibitors cause major changes in splicing products, almost one-third of which are p53 transcriptional targets. Moreover, for DIs, the level of gene expression is controlled through modulation of the splicing rate [27].

### 2.3. SRSF4 Has Diverse Functions in Cells and Tissues

Reverse transcription polymerase chain reaction is a cost-efficient and reliable method for comparing mRNA levels in tissues or cells. The mRNA levels of classic housekeeping genes, such as glyceraldehyde 3-phosphate dehydrogenase* (GAPDH)* and *β*-actin, are not altered across biological replicates [28]. Notably, however, in different stages of testis development [29], typical housekeeping genes, for example,* GAPDH* and *β*-actin, are upregulated in the seminoma comparing with the normal testis [30]. In contrast, SRSF4 expression is not affected by testicular pathologies and may be a good candidate housekeeping gene in this context [31].

Adult-specific exon 10 of the tau gene encodes the microtube binding domain of the tau protein, and SRSF4 regulates exon 10, resulting in increased expression of the abnormal tau isoform FTDD-17, which can lead to neurodegenerative diseases called tauopathies (including Alzheimer's disease, FTDD-17, trisomy 21, and diabetic muscle infarction) [32]. SRSF4 can also interact with pinin, the predominant protein involved in corneal epithelial cell-cell adhesion, and form a multiprotein complex within the nucleus of corneal epithelial cells; SRSF4 may have a role in pinin splicing [33]. Genome-wide analyses confirmed that SRSF4 affects hundreds of gene transcripts and is associated with neural differentiation upon neural induction in P19 cells [34].

## 3. Pathological Functions of SRSF4 in Disease

### 3.1. SRSF4 Is Associated with the Anti-Cisplatin Function of Tumor Cells

*Cis*-Diammineplatinum(II) dichloride (cisplatin), a platinum-based antineoplastic medication, is a chemotherapy medication used to treat a number of cancers. Cisplatin can inhibit DNA replication by covalent binding to DNA [35]. Moreover, cisplatin activates both the DNA damage response (DDR) and phosphatidylinositol 3-kinase (PI3K)/Akt signaling pathways [1]. DDR signaling is involved in PI3K-dependent cell cycle arrest, DNA repair, and cell death by triggering specific and overlapping cascades of signaling events [36].* Trans*-regulatory factors, including SR proteins and hnRNPs, determine alternative splicing of these signaling pathway components through interactions with* cis*-regulatory elements [37].

After treatment of breast cancer cell lines with cisplatin, the expression levels of more than 500 genes are altered, and more than 700 splicing changes occur, as demonstrated by transcriptome analysis; these changes affect the expression of tumor genes involved in tumor cell fate and cause tumor cell death [38]. From inhibitor assays, researchers have shown that cisplatin alters alternative splicing through the PI3K subunit p110*β*, and knocking down SRSF4 can abrogate splicing changes induced by cisplatin and reduce the impact of cisplatin on cell death. Thus, the expression of SRSF4 is related to the therapeutic benefit of cisplatin, and changes in transcripts and splicing related to SRSF4 confer breast cancer cells with anti-cisplatin activity [38].

### 3.2. Single Nucleotide Polymorphisms (SNPs) in SRSF4 Are Related to Nonobstructive Azoospermia (NOA) Susceptibility

Genetic causes of NOA include chromosome mutations, and congenital dysfunctions in spermatogenesis are one cause of male infertility [39]. The pathogenesis of NOA is associated with many susceptibility factors. In the testis, spermatogenesis is accompanied by highly activated transcription and splicing events [40], and SRSFs play key role in splicing during spermatogenesis [41]. Splicing mutation is one reason of sterility; for example, splicing mutations in kelch-like 10 may cause reduced sperm count [42], whereas splicing mutations in zona pellucida-binding protein 1 can lead to sperm head morphological defects [43].

SNPs are single nucleotide mutations at specific positions in the genome. Sixteen SNPs in SRSFs have been shown to be related to a variety of diseases. The SNP rs12046213 (G > A), one of four SNPs in SRSF4, is located 5 kb upstream at 1p35.3 and is significantly associated with NOA susceptibility [44]. Cystic fibrosis transmembrane conductance regulator (CFTR) and microtubule-associated protein tau (TAU) are target proteins of SRSF4 in the testis. CFTR is expressed in germ and Sertoli cells and is involved in spermatogenesis by activating the cyclic adenosine monophosphate-response element binding signaling pathway [41]. TAU, located in the spermatid manchette, promotes microtubule polymerization during spermatid elongation [41]. SRSF4 may affect susceptibility to NOA through interactions with CFTR or TAU [44].

### 3.3. SRSF4 Is Associated with Hematopoietic Progenitor Cell Proliferation

Dyskerin, encoded by the* DKC1* gene, acts as a putative pseudouridine synthase to mediate the posttranscriptional modification of rRNA by conversion of uridine to pseudouridine. Mutations in DKC1 can cause the X-linked form of dyskeratosis congenita (X-DC), a rare progressive congenital disorder with many variable phenotypes (cutaneous pigmentation, premature graying, continuous lacrimation, nail dystrophy, thrombocytopenia, and anemia). X-DC is inherited in an X-linked recessive manner [45, 46], and patients with X-DC exhibit enhanced susceptibility to cancer owing to pseudouridylation of rRNA, which distributes to the pancytopenia and is associated with hypocellularity of the bone marrow [47]. The proliferation rate of hematopoietic progenitors in DKC1 hypomorphic mutant mice is decreased comparing with that in wild-type mice [48].

Stable isotope labeling of amino acids in cell culture is used to detect differences in protein abundance among cells cultured with or without labeled nonradioactive isotopes; all proteins containing isotopic labels are heavier than their counterparts [49]. In previous studies, hematopoietic progenitors were collected from DKC1 hypomorphic mutant mice and wild-type mice, and equal amounts of cells were cultured with or without isotope-labeled amino acid medium. The lysates were then mixed together and analyzed by mass spectrometry. The ratio of the peaks for isotope-labeled or unlabeled proteins was determined by mass spectrometry. SRSF4 is upregulated in wild-type mice compared with that in DKC1 hypomorphic mutant mice; thus, SRSF4 is associated with alternative splicing of genes related to hematopoietic progenitor cell differentiation [48].

### 3.4. SRSF4 Is Implicated in Left Ventricular Hypertrophy

The heart contains cardiomyocytes, fibroblasts, endothelial, and smooth muscle cells. Alternative splicing in cardiomyocytes regulates the expression of diverse proteins and is related to cardiovascular diseases. For example, mutations in myotonic dystrophy type 1 cause inherited neuromuscular disease [50], and mutations in the* RBM20* gene lead to dilated cardiomyopathy [51]. SRSF1 morphants can result in edema in the head and heart [52]. The expression of SRSF4 is decreased in patients with heart failure, and markers of heart failure, that is, brain natriuretic peptide and *β* cardiac myosin heavy chain, are increased in cardiomyocytes in Nkx2.5-Cre transgenic mice with SRSF4 knockout. Moreover, in these mice, RNA-seq analysis showed that SRSF4 knockout altered the expression of genes related to the metabolic pathway, transport, and cytoskeleton organization, suggesting that SRSF4 may be implicated in left ventricular hypertrophy [53].

### 3.5. SRSF4 Participates in the Pathogenesis of Acute Myeloid Leukemia (AML) by Regulating Caspase 8* (CASP8)* Splicing

AML is a type of leukemia that shows abnormal expression of splicing factors [54].* CASP8* plays a central role in programmed cell death as a proapoptotic protease, and* CASP8L* is the main variant in human peripheral blood lymphocytes [55].* CASP8L* exhibits antiapoptotic functions, and its expression is increased CD34+ stem cells of patients with AML-M0 [56]. Compared to healthy controls, the expression of* CASP8L* mRNA is increased and accompanied with the decrease of SRSF4 mRNA expression in new diagnosed AML, the proportion of* CASP8L*/*CASP8A* is also significantly increased, and there is clear correlation between SRSF4 and* CASP8L* mRNA expression; SRSF4 acts as a splicing regulator of* CASP8* and mediates* CASP8* splicing [57].

## 4. Conclusions and Perspectives

Transcriptome complexity and protein diversity are determined by alternative splicing, which is essential for the maintenance of cellular and tissue homeostasis. Additionally, abnormal splicing is related to genetic expression disorders and some diseases [12–14]. SRSFs are critical splicing regulators and are involved in constitutive and alternative splicing. Like other SRSF members, SRSF4 exists in both the nucleus and cytoplasm and is shuttled in and out of the nucleus [23]. SRSF4 plays important roles in RNA metabolism by binding to both exonic and intronic positions. Moreover, SRSF4 can alter gene expression by mediating DI splicing [28] and can affect disease progression by modulating the PI3K/Akt signaling pathway [39]. SRSF4 has also been shown to be associated with AML [57], heart disease [53], reproductive defect disease [45], and proliferation of hematopoietic progenitors [48]. The splicing functions of SRSF4 are implicated in many physiological and pathological processes, and SNPs in SRSF4 may affect the progression of some diseases. Therefore, SRSF4 may have applications as a new therapeutic target.

Inhibition of CLK can alter the phosphorylation of SRSF4 [27]. Therefore, it remains unclear whether CLK is a specific kinase targeting SRSF4. Although early trials have confirmed that SRSF4 is associated with hematopoietic progenitor cell differentiation in mice and pinin splicing in mammalian cells, SRSF4 function in most mammalian cell lines remains unknown. Moreover, studies have been limited by small sample sizes, limited diseases, and shortage of human experimental evidence. Thus, larger, well-designed studies are needed to elucidate the detailed mechanisms of SRSF4 function in diverse diseases. With the application of new technologies, such as second-generation sequencing, our knowledge of the mechanisms of SRSF4 function is expected to improve.
